# Educational Matters

**Published:** 1887-12

**Authors:** 


EDUCATIONAL MATTERS.

In the report of the meeting of the American Dental Society of Europe will be found some severe strictures upon the course of American colleges in granting degrees to unqualified foreigners. The society is composed of representative American dentists, men who are on the ground and should know the status of the American graduates abroad better than even the authorities of the colleges which gave them their diplomas. The report comes from an entirely reliable source, and we have not felt at liberty to suppress it, even were we so disposed. It is presented, therefore, upon their responsibility, and we do not wish to be understood as either endorsing or dissenting from its statements. Every dentist, and especially every graduate, desires to know just what is the sentiment of American dentists abroad upon the subject of education, and to give this information is sufficient reason for presenting the report.

There are very few who will question the fact of the improper conferring of degrees upon foreign candidates in the past. Too much of this has, without doubt, been done, and as a consequence the American degree is discredited in foreign countries, some of them discriminating directly against it. But we are not prepared to charge this upon any particular college or colleges. There has been too lax a sentiment upon the subject on the part of the dental profession in America. The schools have not been held to a sufficiently strict accountability, while reputable students have been content to graduate in the same class with incompetent and disreputable men. We have not, as a body, sufficiently discriminated between the schools which have kept up the standard of qualification and those which have accepted students refused by other institutions.

We believe that within a recent period there has been a marked improvement in the class of student graduates at all our schools, and that the next few years will witness still greater progress. The best of our institutions are, without doubt, ready to go quite as far in the extending of the curriculum as the sentiment of the dental profession will warrant. What is needed is a greater interest in our schools upon the part of practitioners, a better appreciation of what a dental education should be and a higher standard of qualification in the ranks. The schools generally are what the dentists as a body make them, and they fairly reflect the status of the profession. They cannot well rise very much above it, and when dentists demand better schools, we shall have them. We are constantly advancing, and to-day a Delavan disgrace would be impossible. A few years hence some of the schools of to-day will have been either improved or abolished. The calling of public attention to educational affairs will constantly tend to the elevation of public sentiment, and with this end in view we have not hesitated to present the opinions of men who feel deeply upon the subject.

